# The Use of Peptides in the Treatment of Fragile X Syndrome: Challenges and Opportunities

**DOI:** 10.3389/fpsyt.2021.754485

**Published:** 2021-11-04

**Authors:** Alice Romagnoli, Daniele Di Marino

**Affiliations:** Department of Life and Environmental Sciences, New York-Marche Structural Biology Center (NY-MaSBiC), Polytechnic University of Marche, Ancona, Italy

**Keywords:** Fragile X syndrome, targeted therapy, peptides, peptidomimetics, drug development

## Abstract

Fragile X Syndrome (FXS) is the most frequent cause of inherited intellectual disabilities and autism spectrum disorders, characterized by cognitive deficits and autistic behaviors. The silencing of the *Fmr1* gene and consequent lack of FMRP protein, is the major contribution to FXS pathophysiology. FMRP is an RNA binding protein involved in the maturation and plasticity of synapses and its absence culminates in a range of morphological, synaptic and behavioral phenotypes. Currently, there are no approved medications for the treatment of FXS, with the approaches under study being fairly specific and unsatisfying in human trials. Here we propose peptides/peptidomimetics as candidates in the pharmacotherapy of FXS; in the last years this class of molecules has catalyzed the attention of pharmaceutical research, being highly selective and well-tolerated. Thanks to their ability to target protein-protein interactions (PPIs), they are already being tested for a wide range of diseases, including cancer, diabetes, inflammation, Alzheimer's disease, but this approach has never been applied to FXS. As FXS is at the forefront of efforts to develop new drugs and approaches, we discuss opportunities, challenges and potential issues of peptides/peptidomimetics in FXS drug design and development.

## Introduction

Fragile X syndrome (FXS) was first described in 1943 and it is now established as the most common cause of inheritable intellectual disabilities (ID) (1). The molecular cause of FXS is the extensive repeat expansion of a CGG triplet (200 repeats in the full mutation) in the 5′ untranslated region (UTR) and consequential hypermethylation of the *Fmr1* gene, finally leading to transcriptional silencing of the fragile X mental retardation protein (FMRP) (2–4). A small proportion of individuals affected by FXS with different levels of severity show deletion or point mutation in the *Fmr1* gene, that in turn cause the complete loss of FMRP or the production of a functionally deficient protein (5–7).

This disease affects 1:4,000 males and 1:6,000–8,000 females (8, 9) showing symptoms from moderate to severe ID (10). The clinical picture of the syndrome is complex; FXS phenotype displays characteristics in common with autism spectrum disorder (ASD) and attention deficit hyperactivity disorder (ADHD), with general anxiety, social avoidance and hyperactive behaviors (11–13). Seizures, recurrent otitis media, strabismus and obesity are also often occurring in patients affected by FXS. Besides, about 10% of males with FXS display a Prader-Willi like phenotype (14). The physical features of FXS comprehend elongated face, broad forehead, high palate, prominent ears, hyperextensible finger joints, flat feet and macroorchidism (15). All these behavioral, phenotypical and clinical characteristics of FXS, are due to the lack of FMRP, a well-characterized RNA-binding protein, showing crucial functions mainly related to mRNAs metabolism (16). Its main role is represented by the translational repression of numerous key mRNAs in pre- and postsynaptic neurons (17, 18). The FMRP deficiency results in increased protein synthesis, causing the upregulation of several signaling effectors, such as excitatory metabotropic glutamate receptor (mGluR) (19, 20), α-amino-3-hydroxy-5-methyl-4-isoxazole propionic acid (AMPA) receptor (21), extracellular signal-related kinase (ERK1/2) (22–24), matrix metalloproteinase 9 (MMP-9) (25–27), brain-derived neurotrophic factor (BDNF) (28, 29), and mammalian target of rapamycin (mTOR) (30, 31). Moreover, functional impairment in gamma-aminobutyric acid (GABA) receptor and in the endocannabinoid system have been also documented in FXS (32–34). In healthy conditions all these machineries orchestrate neurotransmission and local protein synthesis that impact synaptic plasticity, learning and memory. Hence, in FXS pathology, the lack of FMRP leads to increased protein synthesis with a direct effect on dendritic spine dysgenesis and cognitive disabilities (9, 17), causing the majority of the FXS symptoms. Evidences from *Fmr1* knockout (KO) mice and from human post-mortem brain biopsies showed increased amount and length of dendritic spines, with an immature profile (35–37).

FMRP inhibits translation initiation through its interactions with eIF4E (Eukaryotic translation Initiation Factor 4E) and CYFIP1 (Cytoplasmic FMRP Interacting Protein 1) (4, 7, 38–40). eIF4E is the cap-binding protein known to be activated by the interaction with the scaffold protein eIF4G (Eukaryotic translation Initiation Factor 4G) or inhibited by 4E-binding proteins (4E-BPs), these last being a well-characterized group of proteins that repress protein synthesis (41, 42). The 4E-BPs and eIF4G compete for the same binding site on the eIF4E surface; thus, 4E-BPs inhibit the eIF4E-eIF4G complex formation by sequestering the unbound eIF4E (43, 44). CYFIP1 belongs to the 4E-BPs family and in neurons, mainly at synapses, the FMRP-CYFIP1-eIF4E inhibitory complex regulates protein synthesis during synaptic activity, playing a pivotal role in the modulation of long-term synaptic plasticity at synapses (18, 38). Moreover, the CYFIP1 paralog CYFIP2 is itself able to interact with FMRP and with the FMRP-related proteins FXR1P/2P, which are cytoplasmatic proteins that share with FMRP the functional domains deputed to promote homo- and heteromerization (45, 46).

## Current Strategies in FXS Treatment

Recently, strong effort was dedicated to develop specific FXS pharmacological treatment that can lead to a possible cure, or at least alleviate symptoms (47–51). The most promising or studied treatments for FXS are listed in Table 1. However, although several therapeutic approaches are being tested on different FXS animal models (i.e., *Fmr1* KO mouse, rat and zebrafish; *dFmr* null mutant fly) and patients over the years, an approved and successful curative therapy for FXS is missing to date, and the management of the clinical aspects of the syndrome continue to focus on symptomatic treatment of psychiatric and behavioral problems, rather than the molecular causes (49, 50, 52).

**Table 1:** Treatments for Fragile X syndrome.

**Approach**	**Name**	**Agent class**	**Mechanism of action**
*Fmr1* gene activity restoration	5-azadeoxycytidine (5-azadC)	Chromatin-modifying enzymes inhibitor	Affects DNA methylation levels and epigenetic modifications
		Non-coding RNA (miRNAs and lncRNAs)	Affect DNA methylation state and histones modification
		Viral-vectors or CRISPR-technology	Gene editing or gene replacement
Targeted therapy	AFQ056/Mavoglutarant, Fenobam, MPEP, STX107, CTEP, RO4917523	Group 1 metabotropic glutamate receptors 5 (mGluR5) antagonists	Block mGluRI signaling
	OV101/gaboxadol, Ganaxolone	GABAa and GABAb agonists	Modulate GABA receptors
	Sertraline	Serotonin reuptake inhibitor	Normalizes serotonin and dopamine levels, stimulation of BDNF
	Cannabidiol	Cannabinoid receptors inhibitor	Regulates endocannabinoid signaling pathway
	Lovastatin	RAS signaling inhibitor	Regulates RAS-MAPK-ERK1/2 pathway
	Minocycline	Semi-synthetic tetracycline derivative	Regulates MMP-9 activity
	Metformin	Derivative of guanidine	Normalizes mTOR and MAPK/ERK pathways, phosphorylates eIF4E, and lowers expression of MMP9
	Bay 60–7550, BNP14770, Roflumilast	PDE4, PDE2A, PDE4D inhibitors	Normalize cAMP and cGMP signaling
Symptomatic treatment	Risperidone and aripiprazole	Antipsychotics	Impacts dopaminergic and serotonergic neurotransmission to treat irritability

One of the first approaches that was suggested for FXS treatment was the *Fmr1* gene activity restoration through changes in the DNA methylation levels and epigenetic modifications (53, 54). Although different compounds were tested and successfully achieving *in vitro* reactivation of the *Fmr1* gene, such as 5-azadeoxycytidine (5-azadC) (53), this strategy has not been tested with *in vivo* studies due to safety problems related to low reactivity and high toxicity of these chromatin-modifying enzymes inhibitors (55). Similarly, another strategy involves the use of non-coding RNAs to affect DNA methylation state and histones modification (56). Based on the promising results obtained in cancer and other diseases (57), several miRNAs and lncRNAs were identified and tested in different FXS models (58–60), but their potential use in clinical therapy is still far away similarly to the modern application of gene therapy methods to restore the *Fmr1* gene (61). Indeed, independent groups demonstrated the possibility to use viral-vectors or CRISPR-technology, with encouraging results in preclinical FXS models (62–67); however, the clinical application in patients is being debated for several undisclosed questions, as safety and brain-targeted delivery. Regarding this approach it is also important to consider that the reactivation of the *Fmr1* mRNA with the full mutation could be toxic, as it was demonstrated by a correlation detected between the *Fmr1* mRNA levels in blood and more severe autism features (68).

However, since several compounds are used to treat behavioral and mental problems, such as stimulants or antipsychotics (69), the majority of pharmacological efforts are employed to compensate the absence of FMRP. Among targeted treatment for FXS, several focused on the neurotransmission imbalance associated with FXS. Particular attention has been dedicated in testing the group 1 metabotropic glutamate receptors 5 (mGluR5) antagonists, such as AFQ056/Mavoglutarant, Fenobam, MPEP, STX107, CTEP, RO4917523 (9, 10, 17, 19), and GABA receptors (GABAa and GABAb) agonists (70). In *Fmr1* KO mice these agents showed improvement of several FXS features, including better behavioral abilities, restoration of normal levels of dendritic spines and reduction in protein synthesis (9, 17, 69). Despite these positive results, the transition from animal to human model did not give the same encouraging outcomes, since most of clinical trials failed (71, 72). The high placebo response and the imprecise design and methodology of the trials were the major causes of failure.

Most of drugs tested in FXS pharmacotherapy are compounds already employed or approved for other disorders. Sertraline is a serotonin reuptake inhibitor approved for treating anxiety and mental disabilities in young children and tested in *Fmr1*-KO mouse model. Sertraline normalizes serotonin and dopamine levels, with a rescue on synapse and dendritic formation (73, 74). Even in FXS patients, Sertraline showed favorable results, as several studies demonstrated improvements in language, anxiety and social conduct (75, 76). Cannabidiol (CBD), a synthetic molecule active on cannabinoid receptors, has been used for the treatment of neurological disorders, such as Huntington, Parkinson's and Alzheimer's diseases, but also epilepsy, schizophrenia, autoimmune diseases. All these pathologies have in common altered endocannabinoid signaling pathway, condition confirmed to be deregulated also in FXS animal model (77, 78). Clinical studies indicated good results (79–81), albeit with tolerated side effects. The following FDA-approved drugs have been tested in FXS preclinical and clinical studied: acamprosate (for maintenance of alcohol abstinence), lovastatin (for hypercholesterolemia), minocycline (for acne) and metformin (for non-insulin diabetes mellitus). In particular lovastatin targets the RAS-MAPK-ERK1/2 pathway (82, 83) while minocycline inhibits the MMP-9 activity (25). Both compounds showed promising results in preclinic testing using different model systems, with a reduction in protein synthesis and beneficial cognitive and behavioral aspects (25, 82, 84, 85). Nevertheless, these encouraging data were followed by moderate effects in trials on FXS patients, also expressing the need for a more in-depth investigation on the tolerability of these compounds (86–88). To date, the anti-diabetes drug metformin could be considered as one of the most promising treatments for FXS (89, 90). It has different mechanisms of action, depending on dosage and treatment time, including inhibition of mammalian/mechanistic target of rapamycin complex 1 (mTORC1) and mitogen-activated protein kinase/extracellular signal regulated kinase (MAPK/ERK) pathways, both hyperactivated due to the lack of FMRP in FXS humans and mice (91, 92). As a consequence, metformin also affects the proteins downstream to these cascades, reducing specifically the eIF4E phosphorylation and the translation of MMP-9 (93), which in the pathological condition is the cause for the degradation of proteins essential for synaptic maturation and activity (27). Preclinical studies were performed on *Fmr1*-KO flies and mice models of FXS, showing a rescue of dendritic spine morphology, long-term depression (LTD) of synapses, but also improvement in cognitive, intellectual and social deficits (90, 94). These findings paved the way for treatments in humans, where clinical trials starting in 2018 have been conducted with promising benefits both in terms of behavior and safety of treatment (93, 95–97). Currently 3 trials aimed to evaluate safety, tolerability, and efficacy of metformin in FXS patients are ongoing (ClinicalTrials.gov Identifier: NCT04141163, NCT03862950, NCT03479476). Another interesting therapeutic target in FXS is represented by phosphodiesterases (PDEs), a family of enzymes that regulate the cellular levels of cAMP and cGMP. Among PDEs, PDE1A, PDE2A, and PDE10A have been identified as mRNA targets of FMRP (98). Accordingly, decreased cAMP levels were observed in fly and mouse FXS models and a deregulation of cAMP and cGMP was also identified as a molecular hallmark in FXS patients (99, 100). Hence, several inhibitors have been tested, starting from *Drosophila* model of fragile X, passing through *Fmr1-KO* mice, finally to human trials. Interestingly, inhibiting PDE4 (101, 102), PDE2A (103, 104), PDE4D (105, 106), or synergistically PDE2 and PDE4 (107) demonstrated beneficial effects in terms of rescue of social and behavioral impairments and in dendritic spines morphology in fly and mouse models. Cognitive enhancements were pointed out from FXS trials (105, 106), suggesting that PDEs are candidate targets to develop FXS therapeutic strategy.

## Peptides/Peptidomimetics: a Feasible Strategy for FXS Treatment

All the strategies mentioned so far target different pathways, whose uncontrolled activity seems to be crucial in the pathology of FXS, but also in other neurological disorders and types of cancer (108, 109), leading to pleiotropic effects. Accordingly, the lack of specificity and selectivity, together with bioavailability and safety problems, could be the main drawbacks of these approaches.

In this scenario, a novel and feasible option in FXS pharmacotherapy could be the use of peptides or peptidomimetics.

Since the last 30 years, and especially in the past decade, severe pathologies are being treated with peptides (110) and this class of molecules have attracted the attention of either academia researchers or pharmaceutical industries. Indeed, the global Peptide Therapeutics market reached USD 25.35 billion in 2018 and is expected to achieve USD 50.60 billion by the year 2026. To date, 400–600 peptides are in the preclinical phase of development and more than 60 peptides are FDA-approved (111). The main fields in which therapeutic peptides are currently in development are oncology, metabolic diseases and inflammation (110). Peptides represent an attractive pharmaceutical source due to their excellent properties, namely high selectivity, safety and tolerability (112). However, this kind of approach has never been applied to FXS to restore the imbalance in protein synthesis. Very recently novel structural information opened new possibilities for developing inhibitors acting on the mRNAs translation initiation complex with high specificity and efficiency.

Among these compounds, the 4EGI-1 is one of the most promising inhibitors of the translation activation complex and it has been already tested in different cancer models (113–116). This molecule was also proved to reduce the eIF4E-eIF4G interaction in a FXS mouse model (117). However, the lack of drug-like characteristics, such as poor target specificity and selectivity, high toxicity, several off-targets, severe side effects, poor metabolic stability, poor membrane permeability and rapid proteolysis, make this molecule fairly unsuitable candidates for therapeutic applications.

Furthermore, the putative molecules effective in disrupting the FMRP-CYFIP1-eIF4E or eIF4E-eIF4G complexes formation, are required to target protein-protein interaction (PPI) interfaces, that are large, flat and hydrophobic binding surfaces considered as “undruggable” by small compounds (118–120). One solution could be represented by antibodies, more powerful in targeting PPI, but anyway scarcely able to cross the cell membrane to perform their specific function. In light of this, peptides are now considered as the most appropriate candidates to regulate disease-associated PPIs. However, peptides have intrinsic weak points, and they did not provide encouraging results *in vivo*, likely due to their physical, chemical and structural instability and low membrane permeability (112, 119, 121). To overcome these possible limitations several strategies have been developed, such as amino acids substitution with residues mimicry, termini protection or introduction of chemical modifications aimed at stabilizing their active conformation and increasing cellular permeability (119, 121). These advances in the peptides technology results in the development of an alternative class of compounds called peptidomimetics, that is recently emerging as a class of new potential therapeutic molecules able to target PPIs in the treatment of different pathologies (112). Peptidomimetics are organic molecules with physico-chemical features and structural characteristics comparable with classical oligopeptides, but guarantee enhanced protection against peptidases, improved systemic delivery and cellular uptake, high target specificity and poor immune response (122), and for these reasons their use is under investigation for the treatment of cancer, ischemia, Alzheimer's disease (123–127) and other neurodegenerative disorders (128–131).

On the contrary, the use of peptides/peptidomimetics has never been investigated in the FXS context, but it could represent a viable solution as it might result in a compensation of FMRP absence. Indeed, restoring the FMRP-CYFIP1 deficiency *via* a small chimeric peptide acting on the dysregulation of protein synthesis could be central for the new FXS pharmacological therapy development.

Although the 3D structure of FMRP-CYFIP1 or FMRP with other interacting proteins are still not available, there is a growing number of structures, from different organisms, of complexes belonging to the translation initiation pathway, in particular eIF4G/eIF4E and eIF4E/4E-BPs, and of their regulatory proteins (43, 44, 132, 133). The plethora of structural information, together with the increasing power of computational facilities and refinement of binding prediction tools (110, 112, 119), open the possibility to design peptides/peptidomimetics able to target the translation complexes, with the aim to decrease protein synthesis of specific neuronal mRNAs and rescuing a healthy phenotype in individuals affected by FXS.

**Figure 1:**
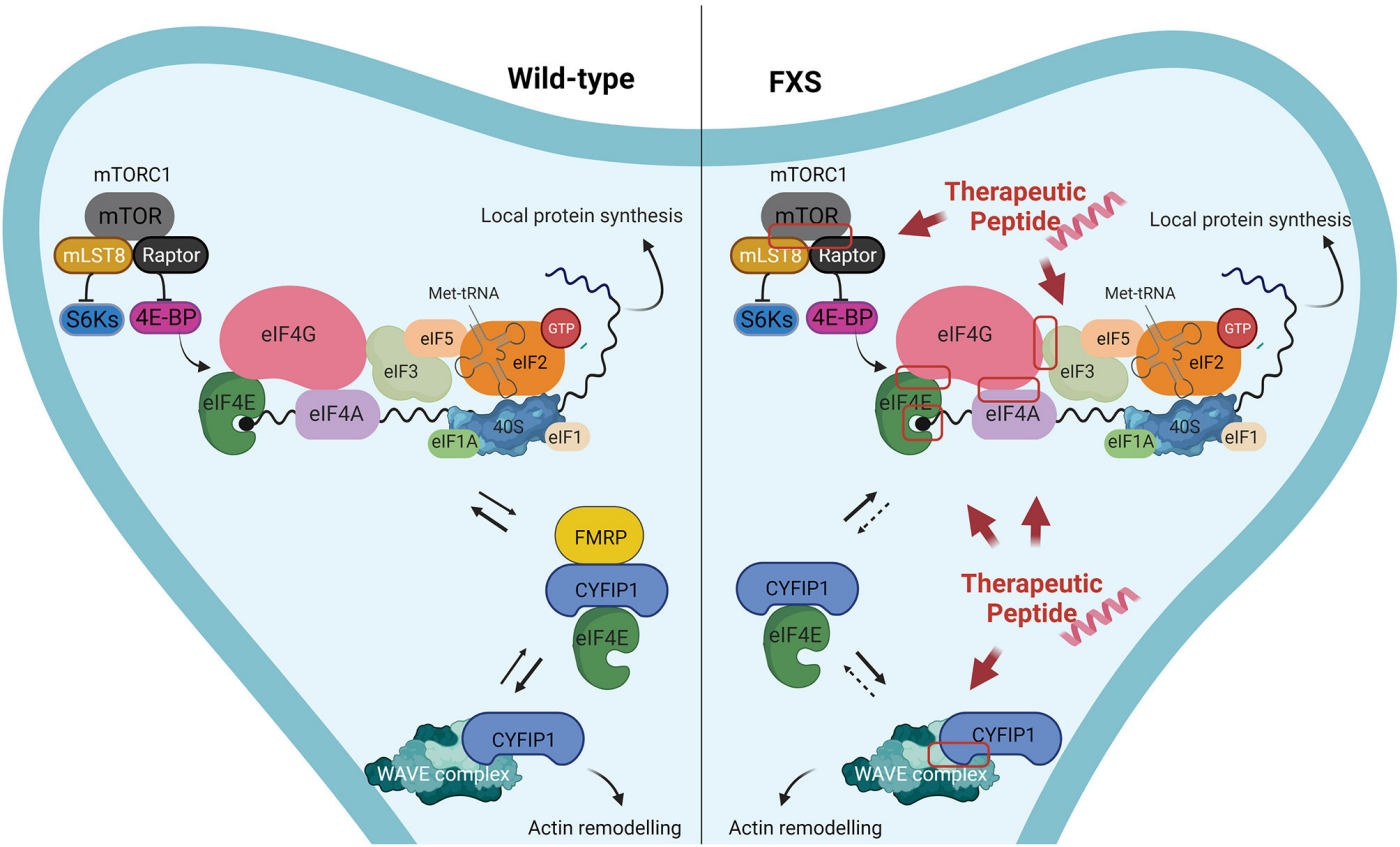
Proposed examples of protein-protein interactions that could be targeted by peptides/peptidomimetics in Fragile X Syndrome. Left panel: In wild-type neurons FMRP plays a key role in down-regulating the translation of FMRP targets, by forming a complex with CYFIP1 and the eukaryotic initiation factor 4E (eIF4E). Furthermore, CYFIP1 can bind and inhibit the WAVE regulatory complex, thereby regulating actin remodeling. Right panel: In FXS neurons the absence of FMRP leads to increased local protein synthesis in neurons, due to the lack of the formation CYFIP1-FMRP-eIF4E complex, that represses the translation initiation. Consequently, a bigger amount of eIF4E is bound to eIF4G, while CYFIP1 interacts mostly with WRC-Rac1-GTP, resulting in higher levels of protein synthesis an altered actin remodeling at dendritic spines. Examples of macromolecular complexes that could be disrupted by peptides/peptidomimetics with the aim to restore the FMRP activity are highlighted by a red box.

For instance, peptides could target and affect the assembly of the eukaryotic Initiation Factor 4F (eIF4F) complex (composed by eIF4E, the DEAD-box helicase eIF4A and the scaffold protein eIF4G) (41, 42), and the formation of 43S pre-initiation complex (43S PIC), composed by small ribosomal subunit 40S and the eukaryotic translation initiation factors (eIFs): eIF1, eIF1A, eIF3, eIF5 (41, 42) (Figure 1). In addition to those already mentioned, the PPIs that could be targeted and disrupted by peptides, could be for instance, eIF4E/cap, eIF4A/eIF4G or eIF4G/eIF3 dimers (Figure 1). Alternatively, peptides/peptidomimetics could be designed to target the eIF4E-upstream regulators, as the PI3K–mTOR pathway, likely inhibiting the interactions of mTORC1 with Raptor and other partners, affecting the activity of the downstream S6K or 4E-BP proteins (Figure 1) (134). Furthermore, not only protein synthesis but also actin dynamic imbalance concurs to the pathophysiology of FXS, leading to defects in dendritic spines morphology (135, 136). Although much information is still lacking, there are indications that Rac1–PAK pathway and FMRP are linked (137, 138). Active Rac1, the Rho-family of small GTPases, activates p21-activated kinases (PAKs) which in turn phosphorylates Cofilin, an actin-binding protein that regulates actin turnover. Rac1 also activates the Wave Regulatory Complex (WRC, composed by five proteins: CYFIP1, NCKAP1, Abi2, HSPC300, and WAVE1) by directly binding CYFIP1 and leading to Arp2/3 complex-mediated actin polymerization (139–141). The release of the X-ray structure of WRC (140), together with data coming from computational analyses that highlighted structural-dynamical features of CYFIP1 (39, 40), may provide useful details to be exploited for the CYFIP1-based peptidomimetics design (Figure 1). To offer some realistic examples, using different regions of CYFIP1 as templates, CYFIP1-derived peptidomimetics could interfere with the eIF4F complex formation by sequestering eIF4E from the binding with eIF4G. Similarly, impeding the CYFIP1/Rac1 interaction or the CYFIP1/NCKAP1 dimer formation, could have a dual beneficial effect in concomitantly restoring normal levels of protein synthesis and actin dynamics, both processes being dysregulated in FXS. Aside from CYFIP1, other 4E-BPs structures in complex with eIF4E are available and represent an attractive template for peptides design. For example, 4E-BP1, 4E-BP2, and Angel1-based peptides were developed and tested in different cancer cell lines (142–144). Furthermore, Lama et al. developed a set of peptides with chemical modifications that increase the pharmacological properties and binding affinity to eIF4E, providing a strong starting point for future oncological preclinical studies (145–147).

On the basis of the knowledge acquired in cancer research, we assume that the peptides/peptidomimetics approach could also be applied to other diseases, in particular in the FXS pharmacotherapeutic.

## Discussion

In the last decade many efforts were employed in research and development of new pharmacological treatments of FXS and simultaneously great advances were made in developing peptides therapeutics against several diseases. With this perspective we speculate that these two roads might cross, starting a new era of the pharmacotherapeutic approach for patients with FXS. To date, chemical compounds that inhibit several pathways deregulated in FXS represent the most studied approaches for FXS management. However, the cause of FXS is a genetic defect (the *Fmr1* silencing) and to effectively correct the absence of FMRP protein is still a challenge. Moreover, although several available treatments are effective in animal models, many clinical trials testified lack of success of these approaches. The emerging peptides technology, in combination with increasingly advanced computational approaches and number of proteins structures deposited in databases, provide an alternative and powerful method to develop a more specific and safe molecules targeting those protein complexes that could be considered as the major players in the FXS pathology. The main point of strength of the use of peptides include selectivity, tolerability, predictable metabolism, ability to target PPIs and lower synthesis complexity that in turn leads to lower production costs compared to others pharmaceutical molecules (119, 121). However, as mentioned above, peptides in general, and thus their possible application against FXS, have several weaknesses that is necessary to discuss. Poor *in vivo* stability, membrane impermeability, and toxicity are widely accounted as major drawbacks in peptides technology. Nevertheless, several of these aspects have been successfully sorted out over recent years through the new technologies available in peptides design field: new bioinformatics tools in combination with other approaches such as virtual screening, structure-based drug design, high throughput screening (HTS) and chemical strategies, provide a comprehensive pharmacological description of putative peptides, improving their chemical and physical features (119, 121, 148). Moreover, the treatment of neurodevelopmental disorders, as FXS, require the delivery of molecules to the brain, accounting for the crossing of the blood–brain barrier (BBB), that could limit the access of peptides to the central nervous system (CNS). In this respect, a strategy to overcome these issues could be the exploration of other drugs administration methods, e.g., the non-invasive intranasal delivery (129, 131). The direct access to CNS allows to overcome limitations linked to the degradation, bioavailability problems and also to possible systemic side effects onset that occur if peptides are present in blood vessels after intravenous administration. Additionally, several strategies have been developed to specifically deliver peptides to target regions of the CNS, such as cyclodextrins, PEI or others (129), resulting in a lower dosage of peptides, also decreasing the toxicity issues which have been demonstrated toward eukaryotic cells (149, 150).

Furthermore, peptides therapy would not be a chronic intervention, but these molecules would be administered only in a limited period of time during the 1st years of life of FXS children, when brain is still remodeling, to allow proper formation of the synaptic network.

Hence, we propose that peptides/peptidomimetics could compensate the FMRP deficiency restoring the imbalance of protein synthesis and actin dynamics, suggesting a new and promising strategy for treating FXS.

## Data Availability Statement

The original contributions presented in the study are included in the article/supplementary material, further inquiries can be directed to the corresponding author/s.

## Author Contributions

This perspective was conceived by DD and AR. DD and AR wrote the manuscript. Table and figure were prepared by AR. All authors contributed to the article and approved the submitted version.

## Funding

AR was supported by FRAXA Foundation.

## Conflict of Interest

The authors declare that the research was conducted in the absence of any commercial or financial relationships that could be construed as a potential conflict of interest.

## Publisher's Note

All claims expressed in this article are solely those of the authors and do not necessarily represent those of their affiliated organizations, or those of the publisher, the editors and the reviewers. Any product that may be evaluated in this article, or claim that may be made by its manufacturer, is not guaranteed or endorsed by the publisher.

## References

[1] Martin JP Bell J. A pedigree of mental defect showing sex-linkage. J Neurol Psychiatry. (1943) 6:154–7. 10.1136/jnnp.6.3-4.154 21611430 PMC1090429

[2] Verkerk AJ Pieretti M Sutcliffe JS Fu YH Kuhl DP Pizzuti A. Identification of a gene (FMR-1) containing a CGG repeat coincident with a breakpoint cluster region exhibiting length variation in fragile X syndrome. Cell. (1991) 65:905–14. 10.1016/0092-8674(91)90397-H 1710175

[3] Oberlé I Rousseau F Heitz D Kretz C Devys D Hanauer A. Instability of a 550-base pair DNA segment and abnormal methylation in fragile X syndrome. Science. (1991) 252:1097–102. 10.1126/science.252.5009.1097 2031184

[4] D'Annessa I Cicconardi F Di Marino D. Handling FMRP and its molecular partners: structural insights into Fragile X Syndrome. Prog Biophys Mol Biol. (2019) 141:3–14. 10.1016/j.pbiomolbio.2018.07.001 30905341

[5] Myrick LK Nakamoto-Kinoshita M Lindor NM Kirmani S Cheng X Warren ST. Fragile X syndrome due to a missense mutation. Eur J Hum Genet. (2014) 22:1185–9. 10.1038/ejhg.2013.311 24448548 PMC4169535

[6] Quartier A Poquet H Gilbert-Dussardier B Rossi M Casteleyn AS Portes V des. Intragenic FMR1 disease-causing variants: a significant mutational mechanism leading to Fragile-X syndrome. Eur J Hum Genet. (2017) 25:423–31. 10.1038/ejhg.2016.204 28176767 PMC5386424

[7] Di Marino D Achsel T Lacoux C Falconi M Bagni C. Molecular dynamics simulations show how the FMRP Ile304Asn mutation destabilizes the KH2 domain structure and affects its function. J Biomol Struct Dyn. (2014) 32:337–50. 10.1080/07391102.2013.768552 23527791

[8] Pugin A Faundes V Santa María L Curotto B Aliaga S Salas I. Clinical, molecular, and pharmacological aspects of FMR1-related disorders. Neurología. (2017) 32:241–52. 10.1016/j.nrleng.2014.10.018 25529181

[9] Hagerman RJ Berry-Kravis E Hazlett HC Bailey DBJ Moine H Kooy RF. Fragile X syndrome. Nat Rev Dis Prim. (2017) 3:17065. 10.1038/nrdp.2017.65 28960184

[10] Richter JD Bassell GJ Klann E. Dysregulation and restoration of translational homeostasis in fragile X syndrome. Nat Rev Neurosci. (2015) 16:595–605. 10.1038/nrn4001 26350240 PMC4688896

[11] Thurman AJ McDuffie A Hagerman R Abbeduto L. Psychiatric symptoms in boys with fragile X syndrome: a comparison with nonsyndromic autism spectrum disorder. Res Dev Disabil. (2014) 35:1072–86. 10.1016/j.ridd.2014.01.032 24629733 PMC4009990

[12] McDuffie A Thurman AJ Hagerman RJ Abbeduto L. Symptoms of autism in males with fragile X syndrome: a comparison to nonsyndromic ASD using current ADI-R scores. J Autism Dev Disord. (2015) 45:1925–37. 10.1007/s10803-013-2013-6 24414079 PMC4096070

[13] Berry-Kravis E Raspa M Loggin-Hester L Bishop E Holiday D Bailey DB. Seizures in fragile X syndrome: characteristics and comorbid diagnoses. Am J Intellect Dev Disabil. (2010) 115:461–72. 10.1352/1944-7558-115.6.461 20945999

[14] Nowicki ST Tassone F Ono MY Ferranti J Croquette MF Goodlin-Jones B. The Prader-Willi phenotype of fragile X syndrome. J Dev Behav Pediatr. (2007) 28:133–8. 10.1097/01.DBP.0000267563.18952.c9 17435464

[15] Salcedo-Arellano MJ Dufour B McLennan Y Martinez-Cerdeno V Hagerman R. Fragile X syndrome and associated disorders: clinical aspects and pathology. Neurobiol Dis. (2020) 136:104740. 10.1016/j.nbd.2020.104740 31927143 PMC7027994

[16] Richter JD Zhao X. The molecular biology of FMRP: new insights into fragile X syndrome. Nat Rev Neurosci. (2021) 22:209–22. 10.1038/s41583-021-00432-0 33608673 PMC8094212

[17] Bagni C Neri G Hagerman R. Fragile X syndrome: causes, diagnosis, mechanisms, and therapeutics. J Clin Invest. (2012) 122:4314–22. 10.1172/JCI63141 23202739 PMC3533539

[18] De Rubeis S Bagni C. Fragile X mental retardation protein control of neuronal mRNA metabolism: insights into mRNA stability. Mol Cell Neurosci. (2010) 43:43–50. 10.1016/j.mcn.2009.09.013 19837168

[19] Bear MF Huber KM Warren ST. The mGluR theory of fragile X mental retardation. Trends Neurosci. (2004) 27:370–7. 10.1016/j.tins.2004.04.009 15219735

[20] Gross C Berry-Kravis EM Bassell GJ. Therapeutic strategies in fragile X syndrome: dysregulated mGluR signaling and beyond. Neuropsychopharmacology. (2012) 37:178–95. 10.1038/npp.2011.137 21796106 PMC3238060

[21] Cheng GR Li XY Xiang YD Liu D Mcclintock SM Zeng Y. The implication of AMPA receptor in synaptic plasticity impairment and intellectual disability in fragile X syndrome. Physiol Res. (2017) 66:715–27. 10.33549/physiolres.933473 28730825

[22] Kim SH Markham JA Weiler IJ Greenough WT. Aberrant early-phase ERK inactivation impedes neuronal function in fragile X syndrome. Proc Natl Acad Sci USA. (2008) 105:4429–34. 10.1073/pnas.0800257105 18332424 PMC2393788

[23] Matic K Eninger T Bardoni B Davidovic L Macek B. Quantitative phosphoproteomics of Murine Fmr1 -KO cell lines provides new insights into FMRP-dependent signal transduction mechanisms. J Proteome Res. (2014) 13:4388–97. 10.1021/pr5006372 25168779

[24] Curia G Gualtieri F Bartolomeo R Vezzali R Biagini G. Resilience to audiogenic seizures is associated with p-ERK1/2 dephosphorylation in the subiculum of Fmr1 knockout mice. Front Cell Neurosci. (2013) 7:46. 10.3389/fncel.2013.00046 23630463 PMC3635025

[25] Bilousova TV Dansie L Ngo M Aye J Charles JR Ethell DW. Minocycline promotes dendritic spine maturation and improves behavioural performance in the fragile X mouse model. J Med Genet. (2009) 46:94–102. 10.1136/jmg.2008.061796 18835858

[26] Dansie LE Phommahaxay K Okusanya AG Uwadia J Huang M Rotschafer SE. Long-lasting effects of minocycline on behavior in young but not adult fragile X mice. Neuroscience. (2013) 246:186–98. 10.1016/j.neuroscience.2013.04.058 23660195 PMC3813005

[27] Michaluk P Wawrzyniak M Alot P Szczot M Wyrembek P Mercik K. Influence of matrix metalloproteinase MMP-9 on dendritic spine morphology. J Cell Sci. (2011) 124:3369–80. 10.1242/jcs.090852 21896646

[28] Kim SW Cho KJ. Activity-dependent alterations in the sensitivity to BDNF-TrkB signaling may promote excessive dendritic arborization and spinogenesis in fragile X syndrome in order to compensate for compromised postsynaptic activity. Med Hypotheses. (2014) 83:429–35. 10.1016/j.mehy.2014.07.007 25113167

[29] Castrén M Lampinen KE Miettinen R Koponen E Sipola I Bakker CE. BDNF regulates the expression of fragile X mental retardation protein mRNA in the hippocampus. Neurobiol Dis. (2002) 11:221–9. 10.1006/nbdi.2002.0544 12460560

[30] Sharma A Hoeffer CA Takayasu Y Miyawaki T McBride SM Klann E. Dysregulation of mTOR signaling in fragile X syndrome. J Neurosci. (2010) 30:694–702. 10.1523/JNEUROSCI.3696-09.2010 20071534 PMC3665010

[31] Price TJ Rashid MH Millecamps M Sanoja R Entrena JM Cervero F. Decreased nociceptive sensitization in mice lacking the Fragile X mental retardation protein: role of mGluR1/5 and mTOR. J Neurosci. (2007) 27:13958–67. 10.1523/JNEUROSCI.4383-07.2007 18094233 PMC2206543

[32] Centonze D Rossi S Mercaldo V Napoli I Ciotti MT De Chiara V. Abnormal striatal GABA transmission in the mouse model for the fragile X syndrome. Biol Psychiatry. (2008) 63:963–73. 10.1016/j.biopsych.2007.09.008 18028882

[33] Curia G Papouin T Séguéla P Avoli M. Downregulation of tonic GABAergic inhibition in a mouse model of fragile X syndrome. Cereb Cortex. (2009) 19:1515–20. 10.1093/cercor/bhn159 18787232 PMC4873279

[34] D'Hulst C De Geest N Reeve SP Van Dam D De Deyn PP Hassan BA. Decreased expression of the GABAA receptor in fragile X syndrome. Brain Res. (2006) 1121:238–45. 10.1016/j.brainres.2006.08.115 17046729

[35] Pan F Aldridge GM Greenough WT Gan WB. Dendritic spine instability and insensitivity to modulation by sensory experience in a mouse model of fragile X syndrome. Proc Natl Acad Sci USA. (2010) 107:17768–73. 10.1073/pnas.1012496107 20861447 PMC2955121

[36] Hinton VJ Brown WT Wisniewski K Rudelli RD. Analysis of neocortex in three males with the fragile X syndrome. Am J Med Genet. (1991) 41:289–94. 10.1002/ajmg.1320410306 1724112

[37] Comery TA Harris JB Willems PJ Oostra BA Irwin SA Weiler IJ. Abnormal dendritic spines in fragile X knockout mice: maturation and pruning deficits. Proc Natl Acad Sci USA. (1997) 94:5401–4. 10.1073/pnas.94.10.5401 9144249 PMC24690

[38] Napoli I Mercaldo V Boyl PP Eleuteri B Zalfa F De Rubeis S. The fragile X syndrome protein represses activity-dependent translation through CYFIP1, a new 4E-BP. Cell. (2008) 134:1042–54. 10.1016/j.cell.2008.07.031 18805096

[39] Di Marino D Chillemi G De Rubeis S Tramontano A Achsel T Bagni C. MD and docking studies reveal that the functional switch of CYFIP1 is mediated by a butterfly-like motion. J Chem Theory Comput. (2015) 11:3401–10. 10.1021/ct500431h 26575774

[40] Di Marino D D'Annessa I Tancredi H Bagni C Gallicchio E. A unique binding mode of the eukaryotic translation initiation factor 4E for guiding the design of novel peptide inhibitors. Protein Sci. (2015) 24:1370–82. 10.1002/pro.2708 26013047 PMC4570532

[41] Sonenberg N Hinnebusch AG. Regulation of translation initiation in eukaryotes. Cell. (2009) 136:731–45. 10.1016/j.cell.2009.01.042 19239892 PMC3610329

[42] Merrick WC Pavitt GD. Protein synthesis initiation in eukaryotic cells. Cold Spring Harb Perspect Biol. (2018) 10:1–22. 10.1101/cshperspect.a033092 29735639 PMC6280705

[43] Grüner S Weber R Peter D Chung MY Igreja C Valkov E. Structural motifs in eIF4G and 4E-BPs modulate their binding to eIF4E to regulate translation initiation in yeast. Nucleic Acids Res. (2018) 46:6893–908. 10.1093/nar/gky542 30053226 PMC6061780

[44] Igreja C Peter D Weiler C Izaurralde E. 4E-BPs require non-canonical 4E-binding motifs and a lateral surface of eIF4E to repress translation. Nat Commun. (2014) 5:1–14. 10.1038/ncomms5790 25179781 PMC4164784

[45] Siomi MC Siomi H Sauer WH Srinivasan S Nussbaum RL Dreyfuss G. FXR1, an autosomal homolog of the fragile X mental retardation gene. EMBO J. (1995) 14:2401–8. 10.1002/j.1460-2075.1995.tb07237.x 7781595 PMC398353

[46] Schenck A Bardoni B Moro A Bagni C Mandel JL. A highly conserved protein family interacting with the fragile X mental retardation protein (FMRP) and displaying selective interactions with FMRP-related proteins FXR1P and FXR2P. Proc Natl Acad Sci USA. (2001) 98:8844–9. 10.1073/pnas.151231598 11438699 PMC37523

[47] Erickson CA Kaufmann WE Budimirovic DB Lachiewicz A Haas-Givler B Miller RM. Best practices in fragile X syndrome treatment development. Brain Sci. (2018) 8:1–9. 10.3390/brainsci8120224 30558274 PMC6315698

[48] Davenport MH Schaefer TL Friedmann KJ Fitzpatrick SE Erickson CA. Pharmacotherapy for fragile X syndrome: progress to date. Drugs. (2016) 76:431–45. 10.1007/s40265-016-0542-y 26858239

[49] Bagni C Oostra BA. Fragile X syndrome: from protein function to therapy. Am J Med Genet Part A. (2013) 161A:2809–21. 10.1002/ajmg.a.36241 24115651

[50] Berry-Kravis EM Lindemann L Jønch AE Apostol G Bear MF Carpenter RL. Drug development for neurodevelopmental disorders: lessons learned from fragile X syndrome. Nat Rev Drug Discov. (2018) 17:280–99. 10.1038/nrd.2017.221 29217836 PMC6904225

[51] Protic D Salcedo-Arellano MJ Dy JB Potter LA Hagerman RJ. New targeted treatments for fragile X syndrome. Curr Pediatr Rev. (2019) 15:251–8. 10.2174/1573396315666190625110748 31241016 PMC6930353

[52] Castagnola S Bardoni B Maurin T. The search for an effective therapy to treat fragile X syndrome : dream or reality ? Front Synaptic Neurosci. (2017) 9:15. 10.3389/fnsyn.2017.00015 29163124 PMC5681520

[53] Chiurazzi P Pomponi MG Willemsen R Oostra BA Neri G. *In vitro* reactivation of the FMR1 gene involved in fragile X syndrome. Hum Mol Genet. (1998) 7:109–13. 10.1093/hmg/7.1.109 9384610

[54] Chiurazzi P Pomponi MG Pietrobono R Bakker CE Neri G Oostra BA. Synergistic effect of histone hyperacetylation and DNA demethylation in the reactivation of the FMR1 gene. Hum Mol Genet. (1999) 8:2317–23. 10.1093/hmg/8.12.2317 10545613

[55] Tabolacci E Palumbo F Nobile V Neri G. Transcriptional reactivation of the FMR1 gene. A possible approach to the treatment of the fragile X syndrome. Genes. (2016) 7:49. 10.3390/genes7080049 27548224 PMC4999837

[56] Holoch D Moazed D. RNA-mediated epigenetic regulation of gene expression. Nat Rev Genet. (2015) 16:71–84. 10.1038/nrg3863 25554358 PMC4376354

[57] Rupaimoole R Slack FJ. MicroRNA therapeutics: towards a new era for the management of cancer and other diseases. Nat Rev Drug Discov. (2017) 16:203–22. 10.1038/nrd.2016.246 28209991

[58] Suhl JA Muddashetty RS Anderson BR Ifrim MF Visootsak J Bassell GJ. A 3' untranslated region variant in FMR1 eliminates neuronal activity-dependent translation of FMRP by disrupting binding of the RNA-binding protein HuR. Proc Natl Acad Sci USA. (2015) 112:E6553–61. 10.1073/pnas.1514260112 26554012 PMC4664359

[59] Wang C Ge L Wu J Wang X Yuan L. MiR-219 represses expression of dFMR1 in Drosophila melanogaster. Life Sci. (2019) 218:31–7. 10.1016/j.lfs.2018.12.008 30528775

[60] Pastori C Peschansky VJ Barbouth D Mehta A Silva JP Wahlestedt C. Comprehensive analysis of the transcriptional landscape of the human FMR1 gene reveals two new long noncoding RNAs differentially expressed in Fragile X syndrome and Fragile X-associated tremor/ataxia syndrome. Hum Genet. (2014) 133:59–67. 10.1007/s00439-013-1356-6 24005575 PMC3898532

[61] Shitik EM Velmiskina AA Dolskiy AA Yudkin DV. Reactivation of FMR1 gene expression is a promising strategy for fragile X syndrome therapy. Gene Ther. (2020) 27:247–53. 10.1038/s41434-020-0141-0 32203197

[62] Gantois I Bakker CE Reyniers E Willemsen R D'Hooge R De Deyn PP. Restoring the phenotype of fragile X syndrome: insight from the mouse model. Curr Mol Med. (2001) 1:447–55. 10.2174/1566524013363492 11899089

[63] Zeier Z Kumar A Bodhinathan K Feller JA Foster TC Bloom DC. Fragile X mental retardation protein replacement restores hippocampal synaptic function in a mouse model of fragile X syndrome. Gene Ther. (2009) 16:1122–9. 10.1038/gt.2009.83 19571888 PMC2741536

[64] Gholizadeh S Arsenault J Xuan ICY Pacey LK Hampson DR. Reduced phenotypic severity following adeno-associated virus-mediated Fmr1 gene delivery in fragile X mice. Neuropsychopharmacol. (2014) 39:3100–11. 10.1038/npp.2014.167 24998620 PMC4229583

[65] Xie N Gong H Suhl JA Chopra P Wang T Warren ST. Reactivation of FMR1 by CRISPR/Cas9-mediated deletion of the expanded CGG-repeat of the fragile X chromosome. PLoS ONE. (2016) 11:e0165499. 10.1371/journal.pone.0165499 27768763 PMC5074572

[66] Park CY Halevy T Lee DR Sung JJ Lee JS Yanuka O. Reversion of FMR1 methylation and silencing by editing the triplet repeats in fragile X iPSC-derived neurons. Cell Rep. (2015) 13:234–41. 10.1016/j.celrep.2015.08.084 26440889

[67] Lee B Lee K Panda S Gonzales-Rojas R Chong A Bugay V. Nanoparticle delivery of CRISPR into the brain rescues a mouse model of fragile X syndrome from exaggerated repetitive behaviours. Nat Biomed Eng. (2018) 2:497–507. 10.1038/s41551-018-0252-8 30948824 PMC6544395

[68] Baker EK Arpone M Aliaga SM Bretherton L Kraan CM Bui M. Incomplete silencing of full mutation alleles in males with fragile X syndrome is associated with autistic features. Mol Autism. (2019) 10:21. 10.1186/s13229-019-0271-7 31073396 PMC6499941

[69] Berry-Kravis E Potanos K. Psychopharmacology in fragile X syndrome—present and future. Ment Retard Dev Disabil Res Rev. (2004) 10:42–8. 10.1002/mrdd.20007 14994287

[70] Schaefer TL Davenport MH Erickson CA. Emerging pharmacologic treatment options for fragile X syndrome. Appl Clin Genet. (2015) 8:75–93. 10.2147/TACG.S35673 25897255 PMC4396424

[71] Berry-Kravis E Des Portes V Hagerman R Jacquemont S Charles P Visootsak J. Mavoglurant in fragile X syndrome: results of two randomized, double-blind, placebo-controlled trials. Sci Transl Med. (2016) 8:321ra5. 10.1126/scitranslmed.aab4109 26764156

[72] Youssef EA Berry-Kravis E Czech C Hagerman RJ Hessl D Wong CY. Effect of the mGluR5-NAM basimglurant on behavior in adolescents and adults with fragile X syndrome in a randomized, double-blind, placebo-controlled trial: fragXis phase 2 results. Neuropsychopharmacol. (2018) 43:503–12. 10.1038/npp.2017.177 28816242 PMC5770759

[73] Hanson AC Hagerman RJ. Serotonin dysregulation in fragile X syndrome: implications for treatment. Intractable Rare Dis Res. (2014) 3:110–7. 10.5582/irdr.2014.01027 25606361 PMC4298641

[74] Lim C Hoang ET Viar KE Stornetta RL Scott MM Zhu JJ. Pharmacological rescue of ras signaling, GluA1-dependent synaptic plasticity, and learning deficits in a fragile X model. Genes Dev. (2014) 28:273–89. 10.1101/gad.232470.113 24493647 PMC3923969

[75] Greiss Hess L Fitzpatrick SE Nguyen DV Chen Y Gaul KN Schneider A. A randomized, double-blind, placebo-controlled trial of low-dose sertraline in young children with fragile X syndrome. J Dev Behav Pediatr. (2016) 37:619–28. 10.1097/DBP.0000000000000334 27560971 PMC5039060

[76] Yoo KH Burris JL Gaul KN Hagerman RJ Rivera SM. Low-dose sertraline improves receptive language in children with fragile X syndrome when eye tracking methodology is used to measure treatment outcome. Psychiatry Clin. (2017) 1:1–8. 10.15406/jpcpy.2017.07.00465

[77] Maccarrone M Rossi S Bari M De Chiara V Rapino C Musella A. Abnormal mGlu 5 receptor/endocannabinoid coupling in mice lacking FMRP and BC1 RNA. Neuropsychopharmacology. (2010) 35:1500–9. 10.1038/npp.2010.19 20393458 PMC3055456

[78] Jung KM Sepers M Henstridge CM Lassalle O Neuhofer D Martin H. Uncoupling of the endocannabinoid signalling complex in a mouse model of fragile X syndrome. Nat Commun. (2012) 3:1080. 10.1038/ncomms2045 23011134 PMC3657999

[79] Heussler H Cohen J Silove N Tich N Bonn-Miller MO Du W. A phase 1/2, open-label assessment of the safety, tolerability, and efficacy of transdermal cannabidiol (ZYN002) for the treatment of pediatric fragile X syndrome. J Neurodev Disord. (2019) 11:16. 10.1186/s11689-019-9277-x 31370779 PMC6676516

[80] Bergamaschi MM Queiroz RHC Chagas MHN de Oliveira DCG De Martinis BS Kapczinski F. Cannabidiol reduces the anxiety induced by simulated public speaking in treatment-naïve social phobia patients. Neuropsychopharmacol. (2011) 36:1219–26. 10.1038/npp.2011.6 21307846 PMC3079847

[81] Busquets-Garcia A Gomis-González M Guegan T Agustín-Pavón C Pastor A Mato S. Targeting the endocannabinoid system in the treatment of fragile X syndrome. Nat Med. (2013) 19:603–7. 10.1038/nm.3127 23542787

[82] Osterweil EK Chuang SC Chubykin AA Sidorov M Bianchi R Wong RKS. Lovastatin corrects excess protein synthesis and prevents epileptogenesis in a mouse model of fragile X syndrome. Neuron. (2013) 77:243–50. 10.1016/j.neuron.2012.01.034 23352161 PMC3597444

[83] Muscas M Louros SR Osterweil EK. Lovastatin, not simvastatin, corrects core phenotypes in the fragile X mouse model. eNeuro. (2019) 6:2019. 10.1523/ENEURO.0097-19.2019 31147392 PMC6565377

[84] Cerezo-Guisado MI García-Román N García-Marín LJ Alvarez-Barrientos A Bragado MJ Lorenzo MJ. Lovastatin inhibits the extracellular-signal-regulated kinase pathway in immortalized rat brain neuroblasts. Biochem J. (2007) 401:175–83. 10.1042/BJ20060731 16952276 PMC1698684

[85] Siller SS Broadie K. Neural circuit architecture defects in a Drosophila model of fragile X syndrome are alleviated by minocycline treatment and genetic removal of matrix metalloproteinase. Dis Model Mech. (2011) 4:673–85. 10.1242/dmm.008045 21669931 PMC3180232

[86] Çaku A Pellerin D Bouvier P Riou E Corbin F. Effect of lovastatin on behavior in children and adults with fragile X syndrome: an open-label study. Am J Med Genet Part A. (2014) 164:2834–42. 10.1002/ajmg.a.36750 25258112

[87] Utari A Chonchaiya W Rivera SM Schneider A Hagerman RJ Faradz SMH. Side effects of minocycline treatment in patients with fragile X syndrome and exploration of outcome measures. Am J Intellect Dev Disabil. (2010) 115:433–43. 10.1352/1944-7558-115.5.433 20687826 PMC4031088

[88] Leigh MJS Nguyen DV Mu Y Winarni TI Schneider A Chechi T. A randomized double-blind, placebo-controlled trial of minocycline in children and adolescents with fragile x syndrome. J Dev Behav Pediatr. (2013) 34:147–55. 10.1097/DBP.0b013e318287cd17 23572165 PMC3706260

[89] Gantois I Khoutorsky A Popic J Aguilar-Valles A Freemantle E Cao R. Metformin ameliorates core deficits in a mouse model of fragile X syndrome. Nat Med. (2017) 23:674–7. 10.1038/nm.4335 28504725

[90] Gantois I Popic J Khoutorsky A Sonenberg N. Metformin for treatment of fragile X syndrome and other neurological disorders. Annu Rev Med. (2019) 70:167–81. 10.1146/annurev-med-081117-041238 30365357

[91] Gkogkas CG Khoutorsky A Cao R Jafarnejad SM Prager-Khoutorsky M Giannakas N. Pharmacogenetic inhibition of eIF4E-dependent Mmp9 mRNA translation reverses fragile X syndrome-like phenotypes. Cell Rep. (2014) 9:1742–55. 10.1016/j.celrep.2014.10.064 25466251 PMC4294557

[92] Hou L Antion MD Hu D Spencer CM Paylor R Klann E. Dynamic translational and proteasomal regulation of fragile X mental retardation protein controls mGluR-dependent long-term depression. Neuron. (2006) 51:441–54. 10.1016/j.neuron.2006.07.005 16908410

[93] Dy ABC Tassone F Eldeeb M Salcedo-Arellano MJ Tartaglia N Hagerman R. Metformin as targeted treatment in fragile X syndrome. Clin Genet. (2018) 93:216–22. 10.1111/cge.13039 28436599 PMC6944702

[94] Monyak RE Emerson D Schoenfeld BP Zheng X Chambers DB Rosenfelt C. Insulin signaling misregulation underlies circadian and cognitive deficits in a Drosophila fragile X model. Mol Psychiatry. (2017) 22:1140–8. 10.1038/mp.2016.51 27090306 PMC5071102

[95] Biag HMB Potter LA Wilkins V Afzal S Rosvall A Salcedo-Arellano MJ. Metformin treatment in young children with fragile X syndrome. Mol Genet Genomic Med. (2019) 7:e956. 10.1002/mgg3.956 31520524 PMC6825840

[96] Protic D Aydin EY Tassone F Tan MM Hagerman RJ Schneider A. Cognitive and behavioral improvement in adults with fragile X syndrome treated with metformin-two cases. Mol Genet Genomic Med. (2019) 7:e00745. 10.1002/mgg3.745 31104364 PMC6625129

[97] Proteau-Lemieux M Lacroix A Galarneau L Corbin F Lepage JF Çaku A. The safety and efficacy of metformin in fragile X syndrome: an open-label study. Prog Neuro-Psychopharmacol Biol Psychiatry. (2021) 110:110307. 10.1016/j.pnpbp.2021.110307 33757860

[98] Maurin T Lebrigand K Castagnola S Paquet A Jarjat M Popa A. HITS-CLIP in various brain areas reveals new targets and new modalities of RNA binding by fragile X mental retardation protein. Nucleic Acids Res. (2018) 46:6344–55. 10.1093/nar/gky267 29668986 PMC6158598

[99] Androschuk A He RX Weber S Rosenfelt C Bolduc FV. Stress odorant sensory response dysfunction in drosophila fragile X syndrome mutants. Front. Mol. Neurosci. (2018) 11:242. 10.3389/fnmol.2018.00242 30135642 PMC6092503

[100] Delhaye S Bardoni B. Role of phosphodiesterases in the pathophysiology of neurodevelopmental disorders. Mol Psychiatry. (2021). 10.1038/s41380-020-00997-9 [Epub ahead of print]. 33414502 PMC8589663

[101] Choi CH Schoenfeld BP Weisz ED Bell AJ Chambers DB Hinchey J. PDE-4 inhibition rescues aberrant synaptic plasticity in Drosophila and mouse models of fragile X syndrome. J Neurosci. (2015) 35:396–408. 10.1523/JNEUROSCI.1356-12.2015 25568131 PMC4287155

[102] Gurney ME Nugent RA Mo X Sindac JA Hagen TJ Fox D 3rd. Design and synthesis of selective phosphodiesterase 4D (PDE4D) allosteric inhibitors for the treatment of fragile X syndrome and other brain disorders. J Med Chem. (2019) 62:4884–901. 10.1021/acs.jmedchem.9b00193 31013090 PMC7444661

[103] Maurin T Melancia F Jarjat M Castro L Costa L Delhaye S. Involvement of phosphodiesterase 2A activity in the pathophysiology of fragile X syndrome. Cereb Cortex. (2019) 29:3241–52. 10.1093/cercor/bhy192 30137253

[104] García-Font N Martín R Torres M Oset-Gasque MJ Sánchez-Prieto J. The loss of β adrenergic receptor mediated release potentiation in a mouse model of fragile X syndrome. Neurobiol Dis. (2019) 130:104482. 10.1016/j.nbd.2019.104482 31129085

[105] Gurney ME Cogram P Deacon RM Rex C Tranfaglia M. Multiple behavior phenotypes of the fragile-X syndrome mouse model respond to chronic inhibition of phosphodiesterase-4D (PDE4D). Sci Rep. (2017) 7:14653. 10.1038/s41598-017-15028-x 29116166 PMC5677090

[106] Berry-Kravis EM Harnett MD Reines SA Reese MA Ethridge LE Outterson AH. Inhibition of phosphodiesterase-4D in adults with fragile X syndrome: a randomized, placebo-controlled, phase 2 clinical trial. Nat Med. (2021) 27:862–70. 10.1038/s41591-021-01321-w 33927413

[107] Paes D Xie K Wheeler DG Zook D Prickaerts J Peters M. Inhibition of PDE2 and PDE4 synergistically improves memory consolidation processes. Neuropharmacology. (2021) 184:108414. 10.1016/j.neuropharm.2020.108414 33249120

[108] Bhat M Robichaud N Hulea L Sonenberg N Pelletier J Topisirovic I. Targeting the translation machinery in cancer. Nat Rev Drug Discov. (2015) 14:261–78. 10.1038/nrd4505 25743081

[109] Laplante M Sabatini DM. mTOR signaling in growth control and disease. Cell. (2012) 149:274–93. 10.1016/j.cell.2012.03.017 22500797 PMC3331679

[110] Kaspar AA Reichert JM. Future directions for peptide therapeutics development. Drug Discov Today. (2013) 18:807–17. 10.1016/j.drudis.2013.05.011 23726889

[111] Muttenthaler M King GF Adams DJ Alewood PF. Trends in peptide drug discovery. Nat Rev Drug Discov. (2021) 20:309–25. 10.1038/s41573-020-00135-8 33536635

[112] D'Annessa I Di Leva FS La Teana A Novellino E Limongelli V Di Marino D. Bioinformatics and biosimulations as toolbox for peptides and peptidomimetics design: where are we? Front Mol Biosci. (2020) 7:66. 10.3389/fmolb.2020.00066 32432124 PMC7214840

[113] Moerke NJ Aktas H Chen H Cantel S Reibarkh MY Fahmy A. Small-molecule inhibition of the interaction between the translation initiation factors eIF4E and eIF4G. Cell. (2007) 128:257–67. 10.1016/j.cell.2006.11.046 17254965

[114] Wu M Zhang C Li XJ Liu Q Wanggou S. Anti-cancer effect of cap-translation inhibitor 4EGI-1 in human glioma U87 cells: involvement of mitochondrial dysfunction and ER stress. Cell Physiol Biochem. (2016) 40:1013–28. 10.1159/000453158 27941351

[115] Descamps G Gomez-Bougie P Tamburini J Green A Bouscary D Maïga S. The cap-translation inhibitor 4EGI-1 induces apoptosis in multiple myeloma through Noxa induction. Br J Cancer. (2012) 106:1660–7. 10.1038/bjc.2012.139 22510748 PMC3349175

[116] Romagnoli A Maracci C D'Agostino M Teana A La Di Marino D. Targeting mTOR and eIF4E: a feasible scenario in ovarian cancer therapy. Cancer Drug Resist. (2021) 4:596–606. 10.20517/cdr.2021.20 PMC9094073 35582305

[117] Santini E Huynh TN Longo F Koo SY Mojica E D'Andrea L. Reducing eIF4E-eIF4G interactions restores the balance between protein synthesis and actin dynamics in fragile X syndrome model mice. Sci Signal. (2018) 10:1–28. 10.1126/scisignal.aan0665 29114037 PMC5858943

[118] Tan YS Lane DP Verma CS. Stapled peptide design: principles and roles of computation. Drug Discov Today. (2016) 21:1642–53. 10.1016/j.drudis.2016.06.012 27326912

[119] Lee ACL Harris JL Khanna KK Hong JH. A comprehensive review on current advances in peptide drug development and design. Int J Mol Sci. (2019) 20:2383. 10.3390/ijms20102383 31091705 PMC6566176

[120] Wells JA McClendon CL. Reaching for high-hanging fruit in drug discovery at protein–protein interfaces. Nature. (2007) 450:1001–9. 10.1038/nature06526 18075579

[121] Fosgerau K Hoffmann T. Peptide therapeutics: current status and future directions. Drug Discov Today. (2015) 20:122–8. 10.1016/j.drudis.2014.10.003 25450771

[122] Lenci E Trabocchi A. Peptidomimetic toolbox for drug discovery. Chem Soc Rev. (2020) 49:3262–77. 10.1039/D0CS00102C 32255135

[123] Ryan P Patel B Makwana V Jadhav HR Kiefel M Davey A. Peptides, peptidomimetics, and carbohydrate–peptide conjugates as amyloidogenic aggregation inhibitors for Alzheimer's disease. ACS Chem Neurosci. (2018) 9:1530–51. 10.1021/acschemneuro.8b00185 29782794

[124] Ellert-Miklaszewska A Poleszak K Kaminska B. Short peptides interfering with signaling pathways as new therapeutic tools for cancer treatment. Future Med Chem. (2017) 9:199–221. 10.4155/fmc-2016-0189 28111982

[125] Marqus S Pirogova E Piva TJ. Evaluation of the use of therapeutic peptides for cancer treatment. J Biomed Sci. (2017) 24:21. 10.1186/s12929-017-0328-x 28320393 PMC5359827

[126] Mabonga L Kappo AP. Peptidomimetics: a synthetic tool for inhibiting protein–protein interactions in cancer. Int J Pept Res Ther. (2020) 26:225–41. 10.1007/s10989-019-09831-5

[127] Vercelli A Biggi S Sclip A Repetto IE Cimini S Falleroni F. Exploring the role of MKK7 in excitotoxicity and cerebral ischemia: a novel pharmacological strategy against brain injury. Cell Death Dis. (2015) 6:e1854. 10.1038/cddis.2015.226 26270349 PMC4558515

[128] Baig MH Ahmad K Saeed M Alharbi AM Barreto GE Ashraf GM. Peptide based therapeutics and their use for the treatment of neurodegenerative and other diseases. Biomed Pharmacother. (2018) 103:574–81. 10.1016/j.biopha.2018.04.025 29677544

[129] Meredith ME Salameh TS Banks WA. Intranasal delivery of proteins and peptides in the treatment of neurodegenerative diseases. AAPS J. (2015) 17:780–7. 10.1208/s12248-015-9719-7 25801717 PMC4476983

[130] De la Torre BG Albericio F. Peptide therapeutics 2.0. Molecules. (2020) 25:2293. 10.3390/molecules25102293 32414106 PMC7287585

[131] Chen RPY. From nose to brain: the promise of peptide therapy for Alzheimer's disease and other neurodegenerative diseases. J Alzheimers Dis Parkinsonism. (2017) 7:2–4. 10.4172/2161-0460.1000314

[132] Grüner S Peter D Weber R Wohlbold L Chung MY Weichenrieder O. The structures of eIF4E-eIF4G complexes reveal an extended interface to regulate translation initiation. Mol Cell. (2016) 64:467–79. 10.1016/j.molcel.2016.09.020 27773676

[133] Peter D Weber R Köne C Chung MY Ebertsch L Truffault V. Mextli proteins use both canonical bipartite and novel tripartite binding modes to form eIF4E complexes that display differential sensitivity to 4E-BP regulation. Genes Dev. (2015) 29:1835–49. 10.1101/gad.269068.115 26294658 PMC4573856

[134] Saxton RA Sabatini DM. mTOR signaling in growth, metabolism, and disease. Cell. (2017) 168:960–76. 10.1016/j.cell.2017.02.004 28283069 PMC5394987

[135] Cingolani LA Goda Y. Actin in action: the interplay between the actin cytoskeleton and synaptic efficacy. Nat Rev Neurosci. (2008) 9:344–56. 10.1038/nrn2373 18425089

[136] Irwin SA Galvez R Greenough WT. Dendritic spine structural anomalies in fragile-X mental retardation syndrome. Cereb Cortex. (2000) 10:1038–44. 10.1093/cercor/10.10.1038 11007554

[137] Chen LY Rex CS Babayan AH Kramár EA Lynch G Gall CM. Physiological activation of synaptic Rac>PAK (p-21 Activated Kinase) signaling is defective in a mouse model of fragile X syndrome. J Neurosci. (2010) 30:10977. 10.1523/JNEUROSCI.1077-10.2010 20720104 PMC2929244

[138] Castets M Schaeffer C Bechara E Schenck A Khandjian EW Luche S. FMRP interferes with the Rac1 pathway and controls actin cytoskeleton dynamics in murine fibroblasts. Hum Mol Genet. (2005) 14:835–44. 10.1093/hmg/ddi077 15703194

[139] Chen B Chou HT Brautigam CA Xing W Yang S Henry L. Rac1 GTPase activates the WAVE regulatory complex through two distinct binding sites. Elife. (2017) 6:e29795. 10.7554/eLife.29795 28949297 PMC5614565

[140] Chen Z Borek D Padrick SB Gomez TS Metlagel Z Ismail AM. Structure and control of the actin regulatory WAVE complex. Nature. (2010) 468:533–8. 10.1038/nature09623 21107423 PMC3085272

[141] Rottner K. WAVE regulatory complex. Curr Biol. (2021) 31:R496–552. 10.1016/j.cub.2021.01.086 34033782 PMC8882368

[142] Song YK Guo H Barengo N Naora H. Inhibition of ovarian cancer growth by a tumor-targeting peptide that binds eukaryotic translation initiation factor 4E. Clin Cancer Res. (2009) 15:4336–47. 10.1158/1078-0432.CCR-08-2924 19458052

[143] Masse M Glippa V Saad H Le Bloas R Gauffeny I Berthou C. An eIF4E-interacting peptide induces cell death in cancer cell lines. Cell Death Dis. (2014) 5:e1500–8. 10.1038/cddis.2014.457 25356869 PMC4237268

[144] Gallagher EE Menon A Chmiel AF Deprey K Kritzer JA Garner AL. A cell-penetrant lactam-stapled peptide for targeting eIF4E protein-protein interactions. Eur J Med Chem. (2020) 205:112655. 10.1016/j.ejmech.2020.112655 32739551 PMC7541464

[145] Lama D Liberatore AM Frosi Y Nakhle J Tsomaia N Bashir T. Structural insights reveal a recognition feature for tailoring hydrocarbon stapled-peptides against the eukaryotic translation initiation factor 4E protein. Chem Sci. (2019) 10:2489–500. 10.1039/C8SC03759K 30881679 PMC6385854

[146] Lama D Quah ST Verma CS Lakshminarayanan R Beuerman RW Lane DP. Rational optimization of conformational effects induced by hydrocarbon staples. Sci Rep. (2013) 3:1–10. 10.1038/srep03451 24336354 PMC6506440

[147] Lama D Verma CS. Deciphering the mechanistic effects of eIF4E phosphorylation on mRNA-cap recognition. Protein Sci. (2020) 29:1373–86. 10.1002/pro.3798 31811670 PMC7255503

[148] Gentilucci L Marco R De Cerisoli L. Chemical modifications designed to improve peptide stability : incorporation of non-natural amino acids, pseudo-peptide bonds, and cyclization. Curr Pharm Des. (2010) 16:3185–203. 10.2174/138161210793292555 20687878

[149] Greco I Molchanova N Holmedal E Jenssen H Hummel BD Watts JL. Correlation between hemolytic activity, cytotoxicity and systemic in vivo toxicity of synthetic antimicrobial peptides. Sci Rep. (2020) 10:13206. 10.1038/s41598-020-69995-9 32764602 PMC7414031

[150] Gupta S Kapoor P Chaudhary K Gautam A Kumar R Raghava GPS. Peptide toxicity prediction. Methods Mol Biol. (2015) 1268:143–57. 10.1007/978-1-4939-2285-7_7 25555724

